# Estimating the burden of A(H1N1)pdm09 influenza in Finland during two seasons

**DOI:** 10.1017/S0950268813002537

**Published:** 2013-10-21

**Authors:** M. SHUBIN, M. VIRTANEN, S. TOIKKANEN, O. LYYTIKÄINEN, K. AURANEN

**Affiliations:** 1 Department of Mathematics and Statistics, University of Helsinki, Helsinki, Finland; 2 Department of Vaccination and Immune Protection, National Institute for Health and Welfare, Helsinki, Finland; 3 Department of Infectious Disease Surveillance and Control, National Institute for Health and Welfare, Helsinki, Finland

**Keywords:** A(H1N1)pdm09, Bayesian analysis, influenza, mathematical modelling

## Abstract

In Finland, the pandemic influenza virus A(H1N1)pdm09 was the dominant influenza strain during the pandemic season in 2009/2010 and presented alongside other influenza types during the 2010/2011 season. The true number of infected individuals is unknown, as surveillance missed a large portion of mild infections. We applied Bayesian evidence synthesis, combining available data from the national infectious disease registry with an ascertainment model and prior information on A(H1N1)pdm09 influenza and the surveillance system, to estimate the total incidence and hospitalization rate of A(H1N1)pdm09 infection. The estimated numbers of A(H1N1)pdm09 infections in Finland were 211 000 (4% of the population) in the 2009/2010 pandemic season and 53 000 (1% of the population) during the 2010/2011 season. Altogether, 1·1% of infected individuals were hospitalized. Only 1 infection per 25 was ascertained.

## INTRODUCTION

The first case of A(H1N1)pdm09 influenza virus (‘swine flu’) in Finland was identified on 10 May 2009 [1]. The pandemic season occurred between September 2009 and May 2010, with the major outbreak in October–December 2009. A second season occurred between November 2010 and April 2011, when ‘swine flu’ co-existed with seasonal A and B influenza strains [2]. National vaccination against A(H1N1)pdm09 in Finland was initiated in October 2009, but only after the peak epidemic in the 2009/2010 season was over in the major part of the population [3].

There are good-quality data about the burden of hospitalized infections and severe outcomes with A(H1N1)pdm09 in Finland [3, 4]. However, there remain a number of important questions regarding the impact of A(H1N1)pdm09 in the Finnish population, including the attack rate (infected/susceptible ratio), severity (hospitalization/infection ratio), and the role of vaccination in mitigating the second season. To answer these questions, one needs to know the true number of all infected cases. However, most infections remained unobserved, i.e. surveillance missed a large portion of infections. Underreporting may have occurred for a number of reasons: (1) only a portion of infected individuals exhibited symptoms; (2) only a portion of symptomatic infections sought medical care; (3) samples for confirming the aetiology were not necessarily taken and the sensitivity of laboratory detection may have been low; (4) positive findings may not have been reported to the national registries.

**Fig. 1:**
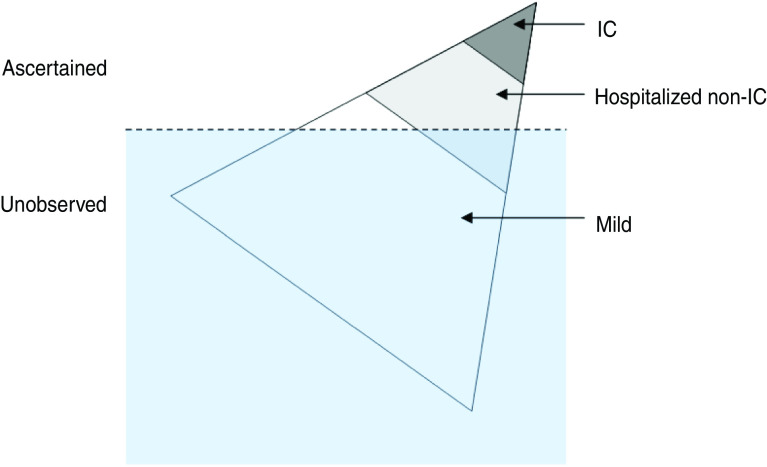
[*colour online*]. The iceberg pyramid of influenza. Infection with influenza can lead to different outcomes with varying severities. The more severe outcomes occur less frequently while being ascertained more easily. The three outcomes in the diagram correspond to the model in this paper. IC, Intensive care.

In general, it was likely that more medical attention was given to individuals with severe forms of disease, leading to biased observations: the probability of ascertaining infection increases with severity (Fig. 1). The ascertainment of infected cases may have varied across regions and age groups, during or between epidemic seasons. For example, the policy of reporting cases changed between the two A(H1N1)pdm09 seasons [3].

Bayesian methodology provides a natural way to handle uncertain data from multiple sources, allowing the combination of heterogeneous evidence [5–7]. In this study we use a Bayesian evidence synthesis to estimate the underlying true incidence of A(H1N1)pdm09 influenza and its severity, based on data on ascertained cases in the first two seasons of A(H1N1)pdm09 in Finland (2009/2010 and 2010/2011). The Methods section describes the surveillance data and the ascertainment model, followed by the Results section, which includes the estimated numbers of A(H1N1)pdm09 infection in Finland over the two seasons as well as estimates of severity and the ascertainment probabilities. The paper concludes with a discussion.

## METHODS

### Surveillance

In Finland (population 5·3 million), the national healthcare system is organized into 20 healthcare districts with catchment populations ranging from 68 000 to 1·4 million, forming five tertiary-care districts. After the first case of pandemic influenza A(H1N1)pdm09 in Finland in 2009, findings positive for A(H1N1)pdm09 were recorded in a specific data collection field in the surveillance system of the National Infectious Disease Registry (NIDR). Notifications concerning the same patient were merged into a single case. In addition to NIDR, a web-based notification system was set up to collate more detailed information on hospitalized cases [3].

#### Hospitalized cases

The data on hospitalized cases from both seasons (2009/2010, 2010/2011) were obtained from the web-based notification system. In the second season, only cases admitted to intensive care (IC) and cases with fatal outcome were recorded in this system. During both seasons, the data on hospitalized cases were specific to A(H1N1)pdm09.

#### Non-hospitalized cases

Numbers of laboratory-confirmed cases with influenza A in the two seasons were obtained from NIDR. In the first season (2009/2010), 99% of influenza A infections were caused by A(H1N1)pdm09 virus, based on virological surveillance and subtyping of viruses [3]. In the second season (2010/2011), more than 95% of influenza A cases in a subsample from a sentinel system were confirmed as A(H1N1)pdm09 [2]. During both seasons, all unidentified influenza A cases were therefore considered as A(H1N1)pdm09 cases and included in the analysis.

All cases included in NIDR and absent in the web-based system were considered as non-hospitalized cases and therefore designated as mild. The data on both the hospitalized and mild cases were stratified by the 20 healthcare districts (regions) and 16 age groups (0–4, 5–9, …, 70–74, >74 years). The population sizes were taken from Statistic Finland (www.stat.fi).

### Vaccination

Altogether 2·6 million A(H1N1)pdm09 vaccine doses were administered in Finland in a special campaign from October 2009 to February 2010. The data regarding vaccinations were retrieved from a nationwide register set up especially for the first A(H1N1)pdm09 season.

The coverage of vaccination varied considerably between different age groups, being highest (81%) in children aged 5–14 years and lowest (32%) in young adults aged 20–29 years [3]. Regional differences in vaccination coverage were less prominent (range 42–61%). In the healthy population aged 3–64 years, the campaign only started a week later than the peak epidemic in the country [3]. For simplicity, we therefore assumed that vaccination effectively took place between the two seasons. The vaccination and demographic data were stratified according to the 20 geographical regions and 16 age groups.

### Definitions and model description

In this paper, infection refers to any infection with A(H1N1)pdm09 that induces protective immunity against subsequent re-infection with the same strain. We focus on the following three infection outcomes: (1) mild infection not requiring hospitalization; (2) infection requiring hospitalization but not admitted to intensive care (hospitalized non-IC); and (3) infections admitted to IC. The two latter are referred to as severe infections. Asymptomatic infections are included in the mild, i.e. non-hospitalized infections, and fatal infections in the IC outcome.

The observed data on A(H1N1)pdm09 suffer from imperfect ascertainment and the numbers of mild cases in particular are only a fraction of the true number of mild infections. Our aim was to estimate the true numbers of individuals with each of the three infection outcomes based on the numbers of ascertained cases and knowledge on the surveillance practice in Finland and epidemics in other countries. We built a model to describe the relationship between the numbers of true infections and ascertained cases in the two A(H1N1)pdm09 seasons (2009/2010, 2010/2011). Bayesian evidence synthesis was used to combine observations with *a priori* knowledge about the unknown model quantities (parameters) to derive a posterior distribution for the parameters. Uncertainties were expressed in terms of probability distributions. The prior distribution expresses this uncertainty about model parameters before including the information from the observations. The posterior distribution describes the uncertainty after including that information.

**Fig. 2:**
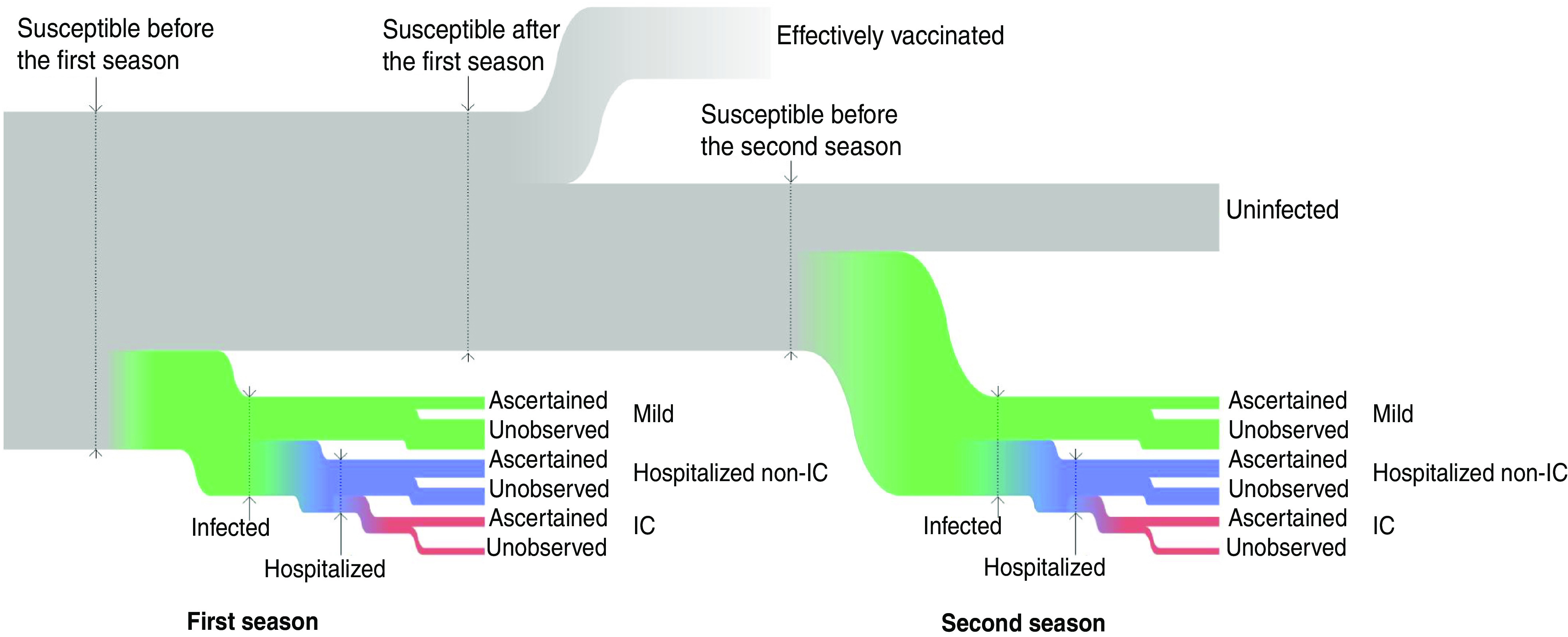
[*colour online*]. The model representation. Susceptible individuals may acquire infection during the first season. Those not infected or protected by vaccination may acquire infection during the second season. Infections are classified as ‘mild’, ‘hospitalized non-intensive care (IC)’ and ‘IC’. Only a fraction of infections are ascertained.

Figure 2 presents the model schematically. We first consider a single epidemic season. Let *p* denote the attack rate, i.e, the probability for a susceptible individual to become infected with A(H1N1)pdm09 during the season. If infected, the individual develops severe disease and is hospitalized with probability *s* (severity or hospitalization/infection ratio), otherwise the infection is termed mild. A hospitalized individual is admitted to IC with probability *g* (IC/hospitalization ratio). Each infection becomes ascertained with a probability which depends on the infection outcome: the ascertainment probabilities for mild, hospitalized non-IC and IC infections are denoted by *α*_*M*_, *α_H_* and *α*_*I*_.

We assume first that each of the six probabilities (*p, s, g, α*_*M*_, *α_I_, α_H_*) is homogeneous across all initially susceptible individuals, *S.* Let *M* denote the total number of mild infections over the epidemic season, *m* of which are ascertained. Similarly, *H* is the total number of hospitalized non-IC individuals (*h* of which are ascertained), and *I* is the total number of IC infections (*i* of which ascertained). We term the vector of the six observables (*M, m, H, h, I, i*) as complete data. Based on the number of susceptible individuals *S* and the complete data, the likelihood function of the six model probabilities is:

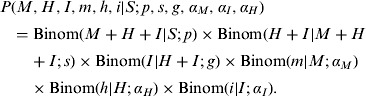
This likelihood is equivalent to one based on a multinomial model for the six observables (see online Supplementary material).

We applied the model simultaneously to data from the two successive influenza seasons (2009/2010, 2010/2011) with separate vectors of observables [*M*^(*j*)^, *m*^(*j*)^, *H*^(*j*)^, *h*^(*j*)^, *I*^(*j*)^, *i*^(*j*)^], *j* = 1, 2. The attack rates *p*^(*j*)^ and the ascertainment probabilities (*α*_*M*_^(*j*)^, *α*_*H*_^(*j*)^, *α*_*I*_^(*j*)^) were allowed to be season-specific, while the severities (*s, g*) were taken to be the same for the two seasons. We assumed that vaccination occurred between the seasons. All individuals infected during the first season were assumed to receive immunity against A(H1N1)pdm09. Others could acquire vaccine-induced immunity with probability *v*. Otherwise, they remained susceptible and could be infected in the second season, i.e. the number of susceptible individuals for the second season *S*^(2)^ was distributed according to a binomial distribution:

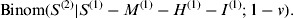


The model was further stratified by allowing some of the probabilities to differ across age strata or geographical regions. In principle, each subpopulation defined by ‘age × region’ could have its own set of the parameters. In practice, we adopted the following assumptions: The probabilities *p*^(*j*)^, *s*, *g*, *α*_*H*_^(*j*)^ were taken to vary across age groups. The probabilities *α*_*M*_^(*j*)^ varied across age groups and regions. The probability *α*_*I*_^(*j*)^ was taken to be the same across all strata. For each of the above probabilities, we defined its average value as the weighted average over age groups and regional strata.

The joint likelihood of the model parameters and unknown observables is:

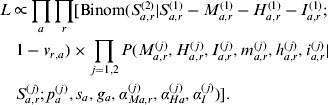
Here *a* and *r* index the age groups and regions.

We assumed there was no pre-existing immunity against A(H1N1)pdm09 before the first season, so the number of susceptible individuals before the first season was equal to the population size in the corresponding stratum *S*_*a,r*_^(1)^=*N*_*a,r*_. We assumed that the vaccines were distributed independently of the infection status of the individuals. We took the probability of receiving vaccine-induced immunity *v*_*a*,*r*_ as a fixed parameter, *v*_*a*,*r*_ = 0·8*V*_*a*,*r*_. Here *V*_*a*,*r*_ is the known vaccine coverage in the corresponding stratum and 0·8 is the vaccine efficacy.

Assuming that vaccination against A(H1N1)pdm09 did not affect the attack rates in those remaining susceptible after the first season, the number of mild infections prevented by vaccination was evaluated as *M*_*a*,*r*_^(2)^*v*_*a*_*_,r_/*(1*−v*_*a,r*_) (see Supplementary material). The prevented numbers of hospitalized and IC cases were estimated similarly.

Table 1 summarizes the prior distributions of the six model parameters. The posterior distribution of the model parameters was explored using a Gibbs sampler [8]. For each of the parameters we present the marginal posterior distribution, the posterior means and 90% credible intervals (CrI). Detailed information about the computational methods is provided in the online Supplementary material.

**Table 1.** Prior distributions

	Parameter	Prior		Rationale
*p*	Attack rate	Beta(2,4)		During previous influenza pandemics in the populations with no pre-existing immunity, the attack rate has been estimated to be up to 50% [9]. Another analysis [10], using computer simulation, estimated the cumulative attack rate during the 2009 pandemic as 31–38% for European countries. A serosurveillance study showed that 33% of children in England were infected in the first season of the A(H1N1)pdm09 epidemic, while the attack rate in other age groups was lower [11].
		Mode: 0·25		
		Mean: 0·33		
		90% PI: 0·86–0·66		
		s.d.: 0·17		
*s*	Severity	Beta(1·33,34)		Most authors define severity as the proportion of hospitalized in all symptomatic infections, while we use the proportion in all infections. The probability of hospitalization for symptomatic infection was estimated [12] to be 0·12–0·26% during the 2009 pandemic, with probability of developing symptoms ∼50%.
		Mode: 0·01		
		Mean: 0·04		
		90% PI: 0·01–0·1		
		s.d.: 0·047		
*g*	IC/hospitalization ratio	Beta(5·3,40)		Estimated [12] as 16%.
		Mode: 0·1		
		Mean: 0·11		
		90% PI: 0·05–0·2		
		s.d.: 0·047		
*α*_M_	Mild case ascertainment probability	Beta(1·33,34)		According to previous studies in similar populations only a small proportion of cases was ascertained: 0·7–2% [13]; 10% [11, 14].
		Mode: 0·01		
		Mean: 0·04		
		90% PI: 0·01–0·1		
		s.d.: 0·047		
*α*_H_	Hospitalized non-IC case ascertainment probability in the first season	Beta(9·6,39)		Assumed to be high. No hospitalized cases were ascertained during the second season.
		Mode: 0·75		
		Mean: 0·71		
		90% PI: 0·5–1		
		s.d.: 0·119		
	In the second season	= 0		
*α*_*I*_	IC case ascertainment probability	= 1		Due to the high attention to IC cases we take *α*_*I*_ = 1.

PI, Probability interval; s.d., standard deviation; IC, intensive care.

Prior distributions were defined for the six model parameters. The mode and spread of each distribution reflect our prior knowledge about the model probabilities. Regarding the prior distribution, a 90% PI means a probability interval within which lies 90% of the distribution.

## RESULTS

**Fig. 3:**
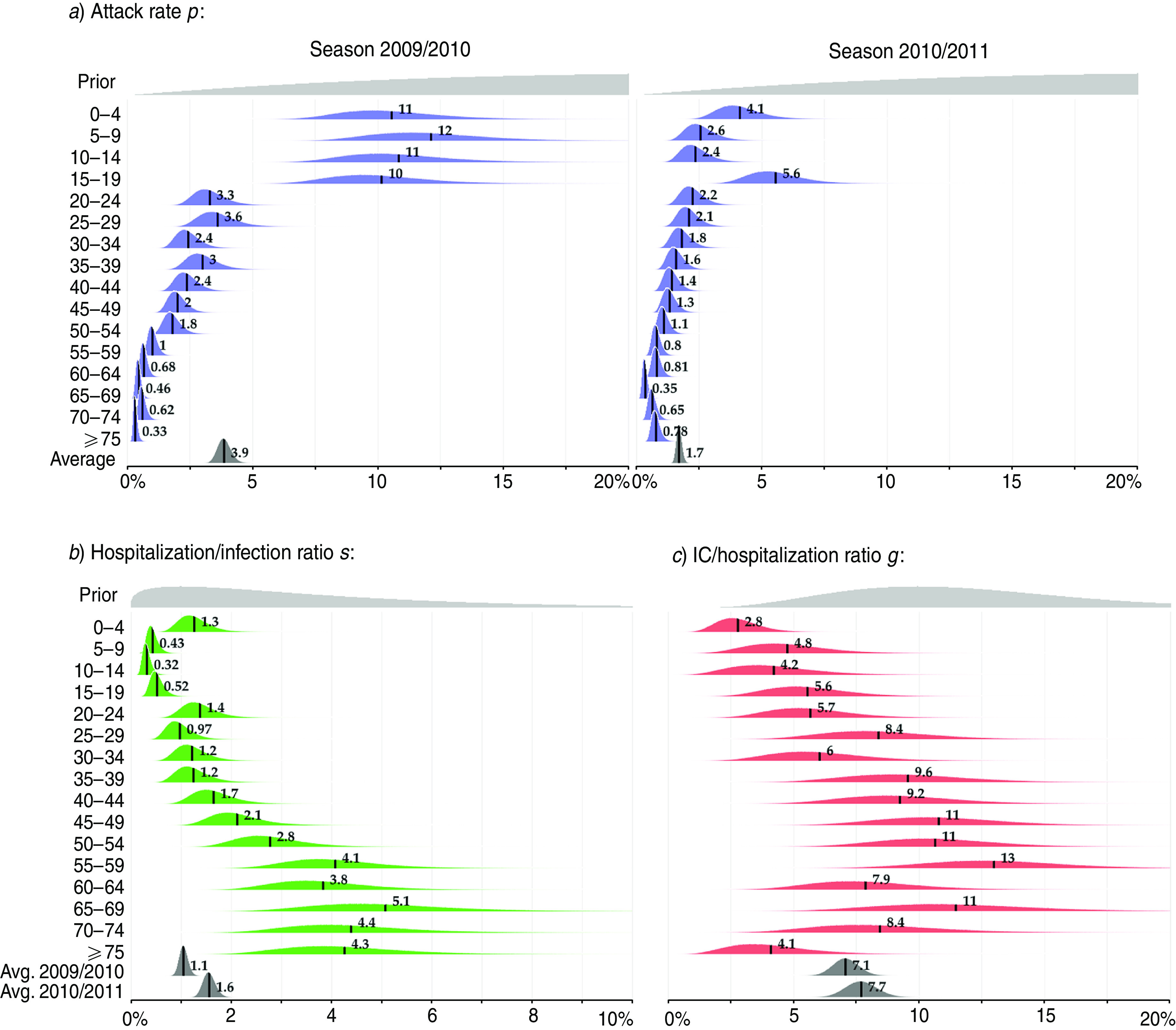
[*colour online*]. The attack rate and severity of A(H1N1)pdm09 influenza. (*a*) The posterior distribution of the attack rate *p* (the infected proportion of the susceptible population) by age group in seasons 2009/2010 and 2010/2011. (*b*) The posterior distribution of severity *s* (hospitalization/infection ratio) by age group. (*c*) The posterior distribution of intensive care (IC) case/hospitalization ratio *g* by age group. The parameters *s* and *g* were assumed to be the same in the two seasons. Their averages were different in two seasons due to different age composition of the infected population. The posterior mean values are highlighted. Note that the scales on the x axes are not the same.

**Fig. 4:**
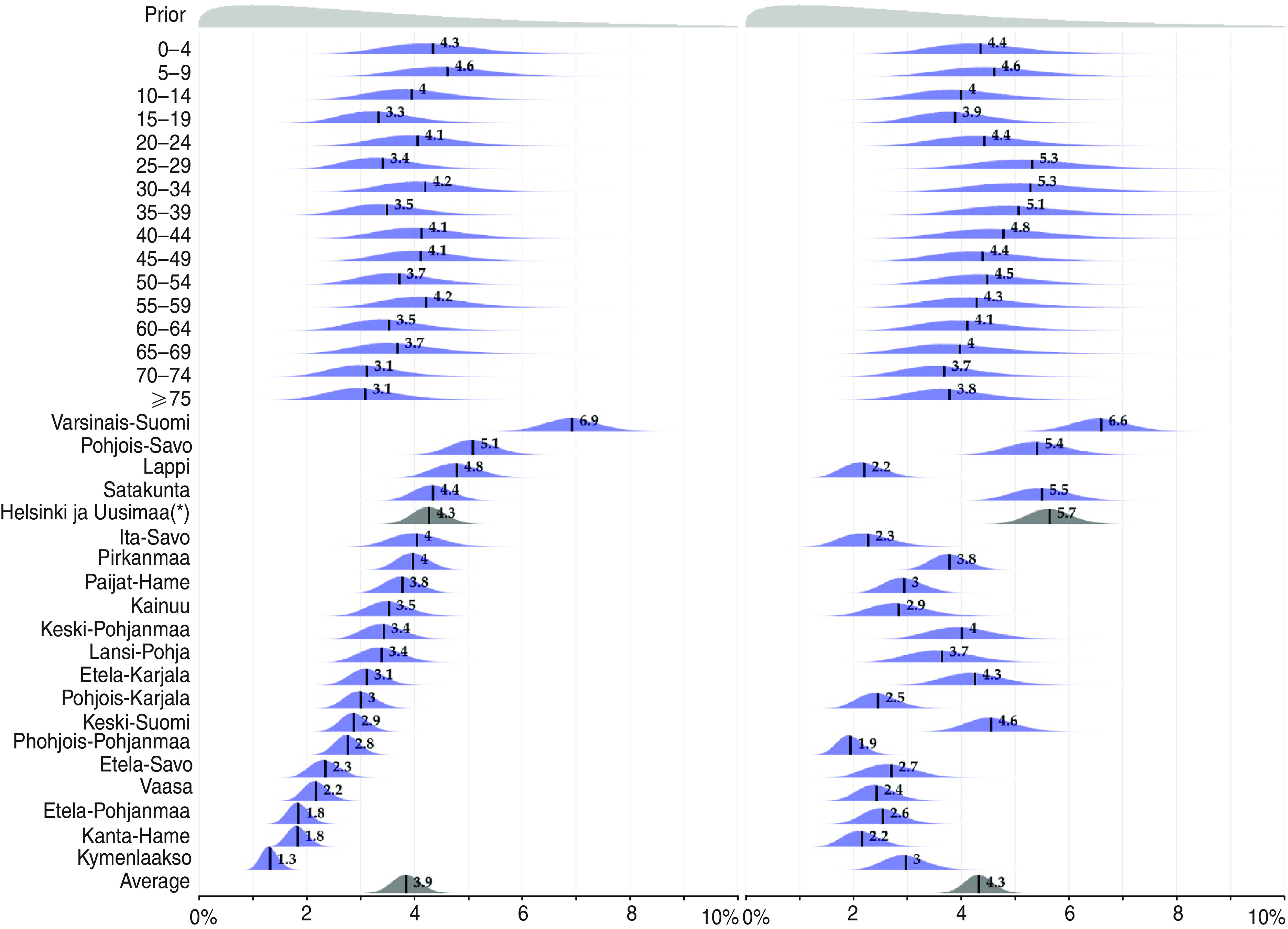
[*colour online*]. The ascertainment probability. The posterior distribution of the ascertainment probability for mild cases *α*_*M*_ by age group and region. The posterior mean values are highlighted. The order of the regions is arbitrary. Helsinki and Uusimaa(*), i.e. the capital region, contains 28% of the population.

Table 2 presents the estimated numbers of infections with different A(H1N1)pdm09 outcomes in the two seasons. The posterior distributions of the model parameters are summarized in Figures 3 and 4.

**Table 2.** The estimated and ascertained numbers of A(H1N1)pdm09 infections during the two seasons (2009/2010 and 2010/2011)

First season (2009/2010)	Stratum	Population			All infections			Hospitalized		IC
		Total		Susceptible	Asc.	Est.	AR	Asc.	Est.	Asc.
		*N*		*S*^(1)^/*N*	*m*^(1)^+*h*^(1)^+*i*^(1)^	*M*^(1)^+*H*^(1)^+*I*^(1)^	*p*^(1)^	*h*^(1)^+*i*^(1)^	*H*^(1)^+*I*^(1)^	*I*^(1)^(≡*i*^(1)^)
	0–4 yr	297 000		100%	1590	31 000	10%	291	386	8
	5–14 yr	587 000		100%	2960	68 200	12%	181	243	5
	15–19 yr	333 000		100%	1200	33 700	10%	128	171	9
	20–29 yr	666 000		100%	1010	23 700	3·6%	191	258	15
	30–64 yr	2 140 000		100%	2430	46 200	2·2%	682	833	83
	⩾65 yr	1 300 000		100%	243	7800	0·6%	134	276	13
	Helsinki	1 820 000		100%	3310	73 100	4·0%	537	730	49
	Turku	692 000		100%	1700	26 300	3·8%	134	205	10
	Tampere	1 230 000		100%	1750	48 300	3·9%	356	484	24
	Kuopio	849 000		100%	1390	32 200	3·8%	284	371	22
	Oulu	733 000		100%	1270	30 700	4·2%	296	375	28
	Total	5 320 000		100%	9420	211 000	3·9%	1610	2170	133
Second season (2010/2011)	Stratum	Population			All infections			Hospitalized		IC
		Total	Vac.	Susceptible	Asc.	Est.	AR	Asc.	Est.	Asc.
		*N*	*V*/*N*	*S*^(2)^/*N*	*m*^(2)^+*h*^(2)^+*i*^(2)^	*M*^(2)^+*H*^(2)^+*I*^(2)^	*p*^(2)^	*h*^(2)^+*i*^(2)^(≡*i*^(2)^)	*H*^(2)^+*I*^(2)^	*I*^(2)^(≡*i*^(2)^)
	0–4 yr	297 000	74%	37%	185	4530	4·2%	0	56	0
	5–14 yr	587 000	81%	31%	182	4580	2·5%	0	17	0
	15–19 yr	333 000	56%	49%	344	9220	5·6%	0	45	0
	20–29 yr	666 000	32%	72%	486	10 700	2·2%	6	120	6
	30–64 yr	2 140 000	53%	64%	919	18 900	1·4%	42	360	42
	⩾65 yr	1 300 000	39%	55%	113	5230	0·73%	4	200	4
	Helsinki	1 820 000	48%	59%	1010	19 200	1·8%	24	290	24
	Turku	692 000	53%	56%	426	6560	1·7%	9	100	9
	Tampere	1 230 000	49%	59%	352	12 600	1·7%	9	190	9
	Kuopio	849 000	57%	52%	298	7690	1·7%	8	120	8
	Oulu	733 000	56%	53%	143	7110	1·8%	2	100	2
	Total	5 320 000	51%	57%	2230	53 100	1·7%	52	800	52

AR, Attack rate; IC, intensive care; Vac., Vaccinated.

Numbers of ascertained cases (Asc.) and the posterior mean estimates of the numbers of infections (Est.) for different outcomes. For the total numbers of infections, the attack rates (*P*) are also given. The proportion of susceptible individuals before the second season is the posterior mean estimate (see Methods section). The results are aggregated into six broad age groups and five tertiary-care districts.

### Attack rate and the burden of illness

Figure 3*a* shows the estimated attack rates (parameter *p*) by age group and season. In the first season (2009/2010), the estimated average attack rate was 3·9% (90% CrI 2·5–4·3), while it was half as much (1·7%, 90% CrI 1·5–1·9) in the second season (2010/2011). The most significant decline in the attack rate was observed in children aged <15 years, whereas in individuals aged ⩾65 years the attack rate was slightly higher in the second season. The number of all A(H1N1)pdm09 infections in the second season corresponds to ∼1% of the total population of Finland.

The attack rate decreased with age in both seasons with a more pronounced trend in the first season: children (<15 years) were 20 times more likely to acquire infection than the elderly (>60 years). In the second season, the corresponding ratio was only 6. The attack rate in teenagers (15–19 years) was noticeably large (5·6%) in the second season.

A total of 211 000 (90% CrI 180 000–230 000) infections were estimated to have occurred in the first season (Table 2). During the second season, an additional 53 000 (90% CrI 47 000–57 000) infections occurred. We estimated that without vaccination 40 000 (90% CrI 35 000–44 000) additional infections would have occurred, including 540 (90% CrI 450–640) hospitalizations and 29 IC infections.

### Severity

The average severity (hospitalization/infection ratio, *s*) in the first season was estimated to be 1% (90% CrI 0·9–1·2), corresponding to 2200 hospitalized cases (90% CrI 2000–2300). In the second season, the average severity was higher (1·6%, 90% CrI 1·4–1·8) because of the older age composition of infected individuals. This corresponded to 800 hospitalized cases (90% CrI 680–925). Taken together, in the two seasons 1·1% of infections were hospitalized. The severity followed a V shape (Fig. 3*b*), with the minimum (0·3%) in those aged 10–14 years, and the two maximums in the youngest (1%) and the oldest (4%) individuals.

The average IC/hospitalization ratio (parameter *g*) was highest (up to 13%) in the general adult population and was lowest in the youngest (3%) and oldest (4%) individuals. There was wide uncertainty in the estimates of this parameter. The average IC/hospitalization ratio was 7% (90% CrI 6–8) in the first season and 8% (90% CrI 7–9) in the second season corresponding to 133 and 52 IC cases, respectively.

### Ascertainment probabilities

Figure 4 shows the posterior distributions of the ascertainment probabilities for mild cases (parameter *α*_*M*_), stratified by age group and region. The weighted average ascertainment probability was estimated to be 3·9% (90% CrI 3·4–4·3) for the first season, i.e. for each ascertained case there were about 24 unobserved infections. The ascertainment probability strongly depended on region but only weakly on age. During the first season the estimates varied from 1·3% to 7%, which corresponds to 14–100 unregistered infections for each ascertained case.

The weighted average ascertainment probability remained almost the same in the second season (4·3%, 90% CrI 3·9–4·8). However, there were notable differences within regions. For example, in Lapland the ascertainment probability during the second season was only half that of the first season. Overall, the regional estimates were more even: from 2% to 6·6%.

The posterior distribution of the ascertainment probability of hospitalized non-IC infections (parameter *α*_*H*_) followed almost exactly the prior distributions in all age groups. During the second season, this parameter was known to be zero as no data were available.

### Posterior uncertainty and sensitivity analyses

There was clear posterior dependence in parameters *p*, *s* and *α*_*M*_ (see Supplementary Fig. S2). For example, the proportion of the ascertained number of mild infections (*m*/*S*) was mainly determined by the product *p* × *α*_*M*_. By combining the prior evidence for these parameters, as well as the data from the several ‘age × region’ strata we were able to identify the marginal posterior intervals for each parameter.

The choice of prior distributions affected the estimates (see Supplementary material). The prior for the ascertainment probability (*α*_*M*_) had the largest effect. Using a less concentrated prior [a beta distribution with the same mode 0·25 but a different standard deviation 0·062 (*vs*. 0·032)] reduced the estimate of the total number of infections over the two seasons to half that of the base-case analysis. On the other hand, using a more localized and shifted prior (mode 0, standard deviation 0·019) doubled the estimate. The prior distributions for the other parameters had less pronounced effects on the estimates. For example, using the uniform prior distribution for the attack rates decreased the estimate of the total number of infections by only 3% (8000 infections) from the base-case analysis. The choice of the prior distributions had no effect on the observed trends in the parameters (e.g. attack rates increasing with age).

The base-case model assumed all parameters (except *α*_*M*_) sharing the same value across the regions. Allowing the attack rate (*p*) to vary by region led to considerably large posterior estimates of the attack rates and the age trend showing in the ascertainment probability (*α*_*M*_). This was probably due to the relatively flat prior on the attack rates. However, these results appear implausible on subjective grounds. Moreover, the Bayesian Information Criterion (BIC) favours the base-case model (26 000 *vs*. 31 000).

## DISCUSSION

We estimated the proportion of the Finnish population infected with pandemic influenza strain A(H1N1)pdm09 during two successive autumn/winter seasons (2009/2010 2010/2011). In the first season, the attack rate was 3·9%, corresponding to 211 000 infected individuals. During the second season, an additional 53 000 infections (1% of the total population) were estimated to have occurred, after accounting for immunity due to vaccination or infection during the first season. Most infections were mild and remained unnotified. In particular, only 4% of infections were estimated to have been ascertained in the national registries. The probability of requiring hospitalization, given influenza infection, was estimated to be ∼1%.

In both seasons, the estimated attack rates decreased markedly with age. This probably reflects the increasing age trend in pre-existing immunity against A(H1N1)pdm09 before the first season, as indicated by the presence of pre-existing antibodies against the virus in serum samples collected in Finland before the present pandemic [1]. In particular, if the numbers of susceptible individuals in older age groups had been smaller, the estimated attack rates would have been larger, while the estimated numbers of cases would have remained about the same.

The average attack rate in the second season was estimated to be half of that in the pandemic (first) season (1·7% *vs*. 3·9%) even when adjusted for protection due to infection or vaccination. This may indicate that it was more difficult for the virus to spread in the partially immune population. The attack rate in teenagers (15–19 years) in the second season was markedly larger than in the other age groups. This pattern reflects the data, in which the number of ascertained cases per population in this age group was significantly larger. Importantly, the proportion vaccinated in this age range was clearly smaller and the estimated proportion of susceptible individuals before the second season was larger than in the younger age group (Table 2). This could explain why the second season attacked teenagers disproportionally.

In the current study, we used 80% vaccine efficacy, irrespective of age group. A lower efficacy would have meant more individuals remained susceptible before the second season, therefore the attack rates in the second season would have been even lower than estimated here. We estimated that vaccination against A(H1N1)pdm09 prevented 40 000 mild, 550 severe, and 29 IC infections in Finland during the second season. This was based on a simplifying assumption that vaccination had no indirect effect on a susceptible individual's probability to acquire infection. However, because the large-scale vaccination probably induced considerable herd immunity, the numbers of prevented cases may have been under-estimated.

We estimated that there were 24 unobserved A(H1N1)pdm09 infections for each ascertained case, with considerable variation across regions (range 14–100). Of note, the average ascertainment probabilities were similar (4%) in both seasons. Nevertheless, it would have been reasonable to assume that underreporting was more common in the second season due to the reduced concern about A(H1N1)pdm09. This would have signified a higher attack rate in the second season. The ascertainment probabilities for mild cases were the only parameters our analysis allowed to vary by region. Consequently, some of the regional variation in the estimated ascertainment rates could be accounted for by regional variation in the actual attack rates.

Given the amount of the data available in this study, the identifiability of the model is admittedly an issue. In particular, some rather informative prior distributions were necessary because otherwise the parameters would have been unidentifiable. We chose relatively weakly informative priors for the attack rates and severities whereas the ascertainment probabilities for mild infection had more informative priors. In addition, to improve the model identifiability, we assumed some shared parameter values, e.g. the age-specific attack rates were taken to be the same across regions.

Although the hospitalization/infection ratio in a given age group was assumed to be the same over the two seasons, the generally older age of infected individuals in the second season led to a higher average severity. There was a clear V shape in the hospitalization/infection ratio with the youngest and the elderly more often requiring hospitalization (Fig. 3*b*). Some previous studies have estimated the death/infection ratio as one of the main aims of inference. In Finland, only a few fatal infections were registered (44 during the first season and 13 during the second), complicating statistical inference on this ratio. A broad definition of A(H1N1)pdm09 infection was used, including mild asymptomatic infection. This was necessary because there were no data on symptomatic cases due to the lack of outpatient data.

A similar analysis using Bayesian methodology estimated that 11% of the population in the UK was infected with A(H1N1)pdm09 over two waves in the 2009 pandemic season [6]. The highest (30%) attack rates were found in the 5–14 years age group. Our estimates of the cumulative incidence of A(H1N1)pdm09 infection in Finland were lower even when accounting for two successive seasons. However, the severity as measured by the hospitalization/infection ratio was actually higher in Finland (all ages: average 1·1% *vs*. 0·19% in the UK). This also translates into a higher per-population risk of hospitalization in Finland (0·06% *vs*. 0·02%). Another study combined serological, virological and epidemiological data from London in a dynamic model leading to even higher estimates of the attack rate (19% in the population, 52% in the 5–14 years age group) [7]. A comparison of pre- and post-pandemic serological surveys yielded an estimated attack rate of 7·6% in the general Dutch population and a much higher (35%) attack rate in children aged 5–19 years [15]. The hospitalization ratio was 0·14%. A meta-analysis of seroepidemiological data collected pre- and post-pandemic in 11 countries suggests that more than 20% of the population in these areas was infected with A(H1N1)pdm09 during the first year of the pandemic [16]. It would have been interesting to repeat such analysis. However, post-pandemic serological data from Finland were not available.

By applying a more rigid prior distribution for the ascertainment probabilities, we would have obtained estimates more similar to those obtained in other studies [6, 7, 15, 16]. However, we believe that the less informative prior better reflected our knowledge about the surveillance in Finland. The estimates of attack rates and severity in our study were based on surveillance data about laboratory-confirmed cases and reasonably vague prior knowledge about the attack rate, severity and the ascertainment probability of mild infections. Improved estimates of the burden and severity of influenza seasons could be obtained if data, e.g. on symptomatic cases and their hospitalization ratio, could be collected. In the meantime, we have demonstrated how the overall burden and severity of influenza outbreaks can be estimated from specific surveillance data in the presence of underreporting and observation biases for severe infections.

## Supplementary Material

Supplementary Material Supplementary information supplied by authors.

Supplementary Material Supplementary information supplied by authors.

Supplementary Material Supplementary information supplied by authors.

Supplementary Material Supplementary information supplied by authors.

## Supplementary material

For supplementary material accompanying this paper visit https://doi.org/10.1017/S0950268813002537. click here to view supplementary material
