# Supplementary figures and images for: Estimating the burden of A(H1N1)pdm09 influenza in Finland during two seasons
Source: Epidemiol Infect. 2013 Oct 21;142(5):964–74. doi: 10.1017/S0950268813002537 (PMC4097990; doi:10.1017/S0950268813002537)

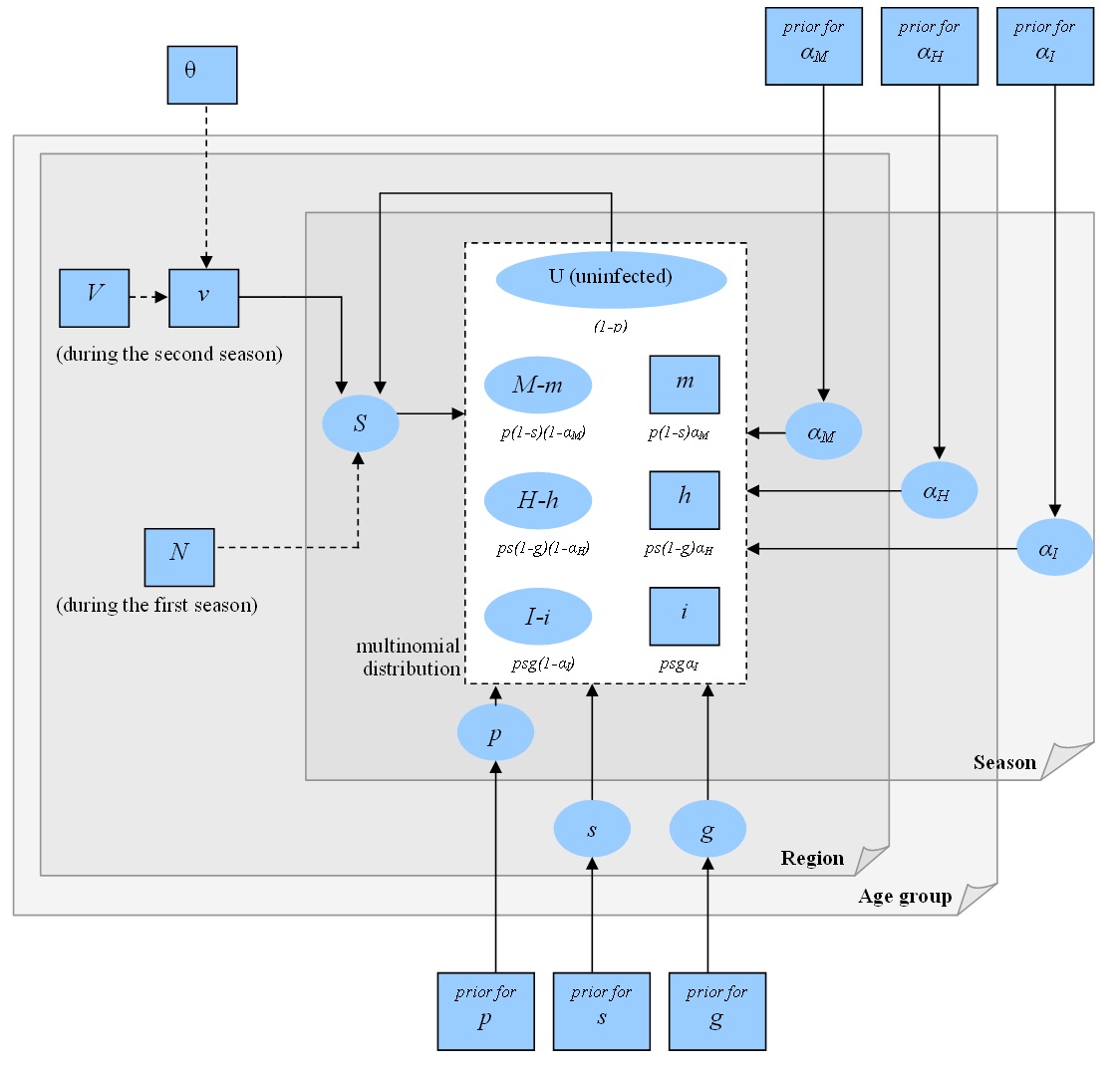

Supplement: Supplementary Material — Supplementary information supplied by authors. [file S0950268813002537sup001.jpg]

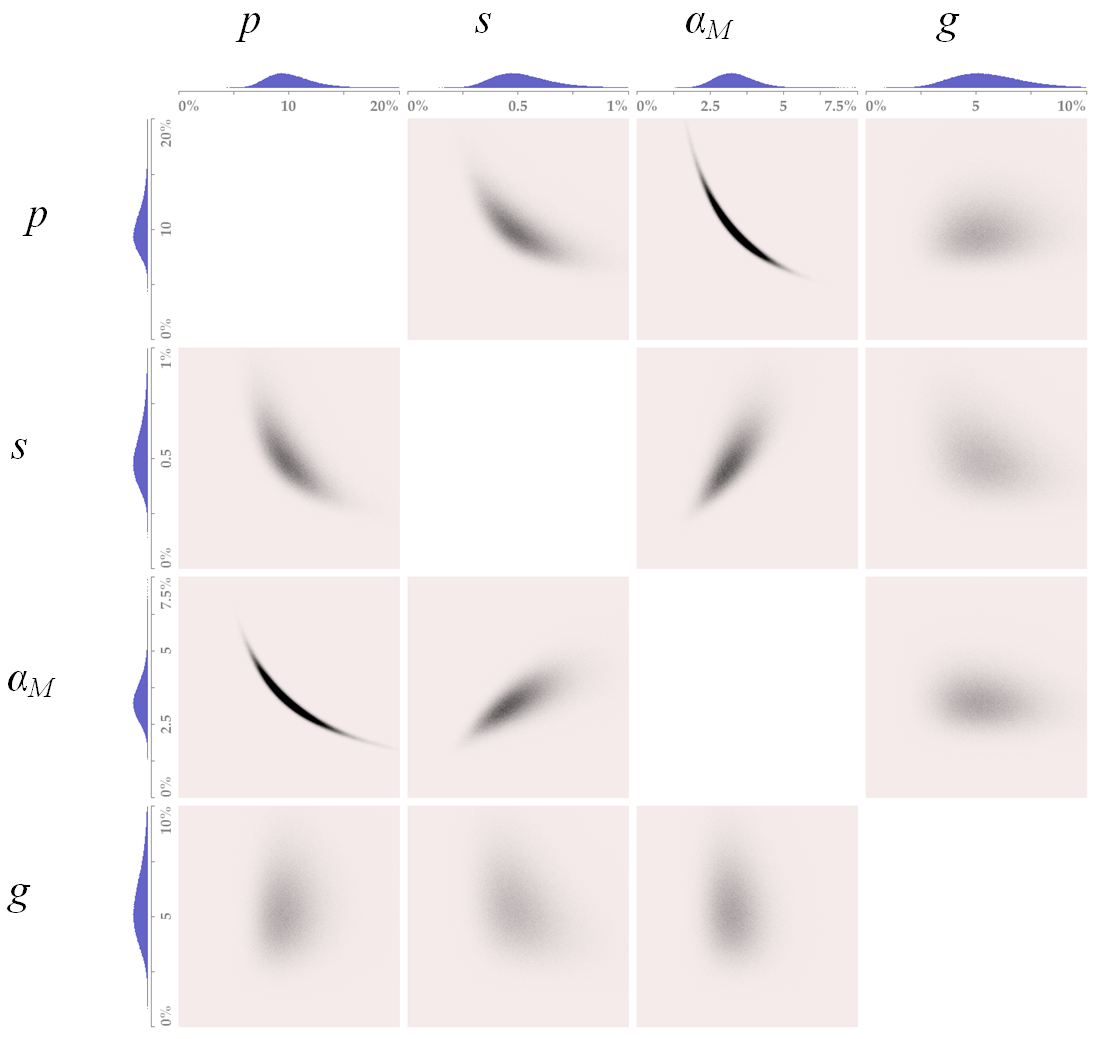

Supplement: Supplementary Material — Supplementary information supplied by authors. [file S0950268813002537sup002.png]
