# Supplementary material for: Estimating the burden of A(H1N1)pdm09 influenza in Finland during two seasons
Source: Epidemiol Infect. 2013 Oct 21;142(5):964–74. doi: 10.1017/S0950268813002537 (PMC4097990; doi:10.1017/S0950268813002537)
Supplement: Supplementary Material — Supplementary information supplied by authors. [file S0950268813002537sup004.doc]

Epidemiology and Infection

Estimating the burden of A(H1N1)pdm09 influenza in Finland during two seasons

M. Shubin, M. Virtanen, S. Toikkanen, O. Lyytikäinen, K. Auranen.

Supplementary Material

**Model representation.**

In the main article, the complete-data likelihood for a given stratum (age group$\times$region) in one season is presented as a product of binomials:

This likelihood is equivalent to a multinomial model with 7 entries (6 free entries) (Figure S1):

$$\begin{matrix} P(M,m,H,h,I,i|S;p,s,g,\alpha_{M},\alpha_{I},\alpha_{H})=\text{Multinom}(U, m, M - m, h, H - h, i, I - i| \\ S; 1 - p, p(1 - s)\alpha_{M}, p(1 - s)(1 - \alpha_{M}), ps(1 - g)\alpha_{H}, ps(1 - g)(1 - \alpha_{H}), psg\alpha_{I}, psg(1 - \alpha_{I})). \end{matrix}$$

Here *U*=*S−M−H−I* stands for uninfected individuals. The equivalence of the two model formulations can be proved using the rule for combining the marginals of the multinomial:

It can be clearly seen from the multinomial formulation that the model has 3 observations and 6 parameters for a single stratum. The parameters define the outcome probabilities in a product form, which explains the hyperbolic posterior dependence betweenpairs of parameters (see Figure S2). However, the use of the Bayesian model with the assumption about the equality of the parameters between different strata helps “borrowing strength" acrossstrata sharing the same age in the parameter estimation, in addition to the impact of the prior distributions.

[figure S1 about here]

**The numbers of prevented cases.**

The expected number of mild infections for a given ‘age group×region’ stratum in the second season is

$$M^{(2)}=S^{(2)}p^{(2)}(1-s)=N(1-p^{(1)})(1-v)p^{(2)}(1-s).$$

The hypothetical number of infections in the absence of vaccination (assuming the vaccination had no effect of attack rate per susceptible in the second season *p*^(2)^) can be estimated as

$$M^{*(2)}=N(1-p^{(1)})p^{(2)}(1-s).$$

Then the number of prevented cases is

$$M^{*(2)}-M^{(2)}=M^{(2)}\left(\frac{M^{*(2)}}{M^{(2)}}-1\right)=M^{(2)}\left(\frac{1}{1-v}-1\right)=M^{(2)}\frac{v}{1-v}$$

The number of prevented hospitalized and IC infections was estimated in the similar way.

**Computational methods.**

The posterior distribution of the model parameters was explored using a Gibbs sampler [1]. Due to the fact that the complete-data likelihood is a product of binomials the full conditional distribution of each of the parameters is a Beta distribution and the full conditional distribution of the other model unknowns (numbers of infections) is a Binomial distribution. For example, the full conditional for probability *s* (hospitalization/infection ratio) is

$$p(s_{a}|*)=\text{Beta}\left(s_{a}|\sum_{r} H_{a,r}+\sum_{r} I_{a,r}+\alpha,\sum_{r} M_{a,r}+\beta\right)$$

here *α* and *β* are the parameters of the prior Beta distribution for *s*. The number of unobserved hospitalized cases *H−h* has the following distribution:

We run the algorithm for 10^7^ iterations recording values at every 10^th^ iteration. The first 1% samples were discarded as “burn-out" iterations. The Gibbs sampler was implemented in Python 2.7.1 with the NumPy external library for samplingfrom random distributions and the Python Image Library (PIL) for visualization.

**Posterior uncertainty.**

Figure S2 presents an example of the joint posterior distribution of parameters *p*, *s*, *α_M_*, *g* in one stratum (Helsinki region, age 15-20 years). There was a clear posterior dependence among the parameters *p*, *s* and *α_M_*. Nevertheless, the peak area of the posterior was still identifiable. Similar patterns were evident in all strata.

[figure S2 about here]

**Sensitivity to the prior distributions.**

Table S1 presents the impact of different prior choices on the estimated number of A(H1N1)pdm09 infections in the two seasons.

[Table S1 about here]

**References**

(1) **Geman S, Geman D**. Stochastic relaxation, gibbs distributions, and the bayesian restoration of images. *IEEE transactions on pattern analysis and machine intelligence* 1984; **6**: 721-741.

Tables and figures

**Fig S1.** The model as a directed acyclic graph. The dotted lines present deterministic relations and the solid lines present stochastic relations. The rectangles and circles represent known and unknown parameters, respectively.

**Fig S2.** Joint posterior distribution of parameters *p*, *s*, *α_M_*, *g* in one stratum (Helsinki region, age 15-20 years)

**Table S1.** The impact of prior distributions on the estimated number of all influenza A(H1N1)pdm09 infection in the two seasons. The results are obtained be changing one prior at a time. SD = standard deviation.

| Parameters | Prior | Mode | Mean | SD | Total |  | Severe |  |
| --- | --- | --- | --- | --- | --- | --- | --- | --- |
| Base-case analysis |  |  |  |  | 258000 |  | 3000 |  |
| Incidence *p* | Beta(2,4) | 0.25 | 0.33 | 0.17 | base case |  |  |  |
|  | Uniform(0,1) | - | 0.50 | 0.28 | -8300 | (-3%) | 0 |  |
|  | Beta(3,6) | 0.28 | 0.33 | 0.14 | +12000 | (+5%) | 0 |  |
| Severity *s* | Beta(1.33,34) | 0.01 | 0.04 | 0.032 | base case |  |  |  |
|  | Uniform(0,1) | - | 0.5 | 0.28 | 0 |  | +50 | (+2%) |
|  | Beta(1.13,15) | 0.01 | 0.07 | 0.06 | 0 |  | +30 | (+1%) |
|  | Beta(1,50) | 0 | 0.02 | 0.02 | +3700 | (+1%) | -60 | (-2%) |
|  | Beta(1,99) | 0 | 0.01 | 0.01 | +8300 | (+3%) | -140 | (-5%) |
| IC/hospitalization ratio *q* | Beta(5.3,40) | 0.1 | 0.11 | 0.047 | base case |  |  |  |
|  | Uniform(0,1) | - | 0.5 | 0.28 | 0 |  | +150 | (+5%) |
|  | Beta(1,9) | 0 | 0.1 | 0.09 | 0 |  | +210 | (+7%) |
|  | Beta(10,90) | 0.09 | 0.1 | 0.029 | 0 |  | -40 | (-4%) |
| Ascertainment | Beta(1.33,34) | 0.01 | 0.04 | 0.032 | base case |  |  |  |
| probability *α_M_* | Uniform(0,1) | - | 0.5 | 0.28 | -234000 | (-90%) | -290 | (-10%) |
| of the mild cases | Beta(1.13,15) | 0.01 | 0.07 | 0.06 | -120000 | (-45%) | -50 | (-2%) |
|  | Beta(1,50) | 0 | 0.02 | 0.02 | +240000 | (+90%) | +30 | (+1%) |
|  | Beta(1,99) | 0 | 0.01 | 0.01 | +700000 | (+270%) | +50 | (+3%) |
| Ascertainment | Beta(9.6,3.9) | 0.75 | 0.71 | 0.119 | base case |  |  |  |
| probability *α_H_* | Uniform(0,1) | - | 0.5 | 0.28 | 0 |  | +180 | (+6%) |
| of the hospitalized cases | Beta(50,1) | 1 | 0.98 | 0.02 | 0 |  | -700 | (-24%) |
|  | Beta(99,1) | 1 | 0.99 | 0.01 | 0 |  | -700 | (-24%) |
